# Synergistic Stimulation with Different TLR7 Ligands Modulates Gene Expression Patterns in the Human Plasmacytoid Dendritic Cell Line CAL-1

**DOI:** 10.1155/2015/948540

**Published:** 2015-12-06

**Authors:** Tobias Hilbert, Folkert Steinhagen, Christina Weisheit, Georg Baumgarten, Andreas Hoeft, Sven Klaschik

**Affiliations:** Department of Anesthesiology and Intensive Care Medicine, University Hospital Bonn, Sigmund-Freud-Strasse 25, 53127 Bonn, Germany

## Abstract

*Objective*. TLR7 ligation in plasmacytoid dendritic cells is promising for the treatment of cancer, allergy, and infectious diseases; however, high doses of ligands are required. We hypothesized that the combination of structurally different TLR7 ligands exponentiates the resulting immune response.* Methods*. CAL-1 (human pDC line) cells were incubated with the TLR7-specific adenine analog CL264 and single-stranded 9.2s RNA. Protein secretion was measured by ELISA. Microarray technique was used to detect modified gene expression patterns upon synergistic stimulation, revealing underlying functional groups and networks. Cell surface binding properties were studied using FACS analysis.* Results*. CL264 in combination with 9.2s RNA significantly enhanced cytokine and interferon secretion to supra-additive levels. This effect was due to a stronger stimulation of already regulated genes (by monostimulation) as well as to recruitment of thus far unregulated genes. Top scoring canonical pathways referred to immune-related processes. Network analysis revealed IL-1*β*, IL-6, TNF, and IFN-*β* as major regulatory nodes, while several minor regulatory nodes were also identified. Binding of CL264 to the cell surface was enhanced by 9.2s RNA.* Conclusion*. Structurally different TLR7 ligands act synergistically on gene expression patterns and on the resulting inflammatory response. These data could impact future strategies optimizing TLR7-targeted drug design.

## 1. Introduction

Immune cells utilize toll-like receptors (TLRs) to sense pathogen associated molecular patterns (PAMPs), which represents the starting point of the innate immune response [1]. Plasmacytoid dendritic cells (pDCs) are at the nexus between innate and adaptive immunity and are thereby key contributors to the host's response to various pathogens. Furthermore, pDCs are the major source of type 1 interferon (IFN) in humans and are, thus, of particular importance for antiviral immunity and autoimmune diseases [2]. In human beings, pDCs and, to a lesser extent, B cells constitutively express TLR7 and TLR9, both of which sense nucleic acids [3–5]. TLR7, originally known to recognize imidazoquinoline derivatives (e.g., imiquimod and resiquimod/R 848) and guanine analogs (e.g., loxoribine), recognizes single-stranded (ss) RNA derived from RNA viruses such as influenza A virus, vesicular stomatitis virus, and human immunodeficiency virus [1]. In pDCs, TLR7 recognizes these ligands in specialized endolysosomes, resulting in the subsequent activation of NF-*κ*B and interferon regulatory factor (IRF) 7 via MyD88, and induces production of inflammatory cytokines and type 1 IFN, respectively [1]. Thus, studying TLR7 in human pDCs is of particular interest. Preclinical studies utilizing TLR7 ligation revealed promising results in the treatment of cancer, allergy, and infectious diseases [6, 7]. However, clinical studies using TLR7 ligands systemically are problematic, as they have been associated with severe side effects such as hematological toxicity [6]. Thus, pharmacological strategies are needed to achieve potent TLR7 responses. One approach is the synergistic use of two compounds. In this study, we hypothesized that the simultaneous use of two structurally and chemically different TLR7 ligands unfolds synergistic effects on human pDCs. To test this hypothesis, we coadministered the adenine analog CL264, a potent and highly specific TLR7 ligand, together with the natural ssRNA 9.2s RNA to the human pDC line CAL-1. Our data may broaden our understanding of TLR7 activation and could impact future strategies optimizing TLR7-targeted drug design.

## 2. Materials and Methods

### 2.1. Materials and Reagents

PBS (pH 7.4) and RPMI 1640 were purchased from Life Technologies (CA, USA). The latter was supplemented with 10% heat inactivated fetal bovine serum (FBS Superior, Biochrom AG, Berlin, Germany) and 2 mmol/L Glutamine (Life Technologies). Cell culture flasks and 24- and 96-well flat-bottom plates were obtained from Greiner Bio-One (Frickenhausen, Germany). CL264 (tlrl-c264e) and FITC-labeled CL264 (tlrl-fc264) were purchased from InvivoGen (CA, USA) and dissolved in sterile, RNAse-free water (Sigma-Aldrich, MO, USA) containing 10% DMSO (Sigma-Aldrich) and further diluted in culture medium. CL264 (formula C_47_H_73_N_13_O_7_S, molecular weight 413.43) is a 9-benzyl-8-hydroxyadenine derivative containing a glycine on the benzyl group (in para). According to the manufacturer specification, the substance we used is highly pure (endotoxin level < 0.001 EU/*µ*g). The oligoribonucleotides (ORNs) 9.2s RNA and the nonstimulatory control Poly-A RNA were individually designed and obtained from Biomers (Ulm, Germany) and also dissolved in sterile, RNAse-free water [8]. Further dilutions were made using culture medium. Poly-L-arginine hydrochloride (P7762) and FITC (F7250) were provided by Sigma-Aldrich. TRIzol reagent was purchased from Life Technologies. Chloroform, isopropanol, and ethanol were obtained from Sigma-Aldrich. DMSO concentration in the cell culture medium never exceeded 0.1 vol%.

Reagents for Fluorescent Activated Cell Sorting (FACS) were purchased from Miltenyi Biotec (Bergisch Gladbach, Germany) and BD Biosciences (CA, USA), respectively. Assay buffer for FACS was prepared by adding 0.5% FBS and 2 mM EDTA (0.5 M, pH 8.0, Merck KGaA, Germany) to PBS. Paraformaldehyde (PFA) was obtained from Sigma-Aldrich.

### 2.2. Culture and Activation of CAL-1 Cells

The human pDC line CAL-1 (kindly provided by Drs. Maeda and Kamihira) was grown in complete medium (RPMI 1640) supplemented with 2 mmol/L Glutamine and 10% heat inactivated FBS and cultured as previously described [9].

For gene expression studies, cells were plated in a 24-well plate (3 × 10^6^ cells in 3 mL per well), and, for ELISA studies, cells were plated in a 96-well plate (2 × 10^5^ cells in 0.2 mL per well). After overnight resting in serum reduced (1% FBS) medium, CAL-1 cells were stimulated with the TLR7 ligand CL264 (5 *µ*g/mL), with 9.2s RNA (2 *µ*g/mL), or with the combination of both. The reagents were diluted in culture medium and added to the cell suspension in the wells of the 96- or 24-well plates, respectively. Vehicle and nonstimulatory Poly-A RNA (2 *µ*g/mL) were used as control. Before stimulation, ORNs were complexed with the polycationic polypeptide poly-L-arginine hydrochloride (PLarg, 2 mg/mL) to facilitate RNA delivery. ORNs were incubated with PLarg for 2 minutes at room temperature before adjusting to the desired concentration by adding culture medium and incubating for another 15 minutes [8]. PLarg alone or 9.2s RNAs not complexed to PLarg were used in control conditions, respectively. After addition of the ligands, the cell density was adjusted to 1 × 10^6^ cells/mL by adding fresh, prewarmed cell culture medium.

After incubating for the indicated periods of time, the supernatant from the 96-well plates was collected and stored at −80°C for later analysis of proinflammatory cytokines and type 1 interferon. From 24-well plates, the cell suspension was carefully aspirated and transferred to a 15 mL conical tube. Cells were sedimented by centrifugation at 2.000 rpm for 5 minutes. The supernatant was discarded and the cell pellet was resuspended in 2 mL TRIzol reagent and stored at −80°C for subsequent extraction of total RNA.

For some secretion experiments, ligands were added sequentially instead of simultaneously with an intermediary wash step. After incubation with a first ligand for 2 hrs, the plate was centrifuged, followed by careful aspiration of the medium. Cells were washed 3 times with prewarmed PBS, each with an intermediary centrifugation step. Subsequently, the other ligand was added in prewarmed cell culture medium, and cells were incubated for additional 6 hrs.

### 2.3. Measurement of Cytokines and IFN-*β* in Cell Culture Supernatant

TNF-*α*, IL-6, and IFN-*β* levels in the cell culture supernatant were quantified using commercially available ELISA kits (BD Biosciences (CA, USA) and PBL Assay Science (NJ, USA), resp.) according to manufacturer instructions.

### 2.4. Isolation of Total RNA

After thawing cells suspended in TRIzol reagent, they were left at room temperature for another 5 minutes and well pipetted up and down to lyse the cell membrane. 400 *µ*L chloroform was added and the suspension was shaken vigorously, followed by incubation at room temperature for 3 minutes. After centrifugation for 15 minutes at 12.000 rpm and +4°C, the upper aqueous phase was carefully aspired and transferred into an RNAse-free tube. The RNA was precipitated using 1 mL isopropanol and incubating at room temperature for 10 minutes. After another centrifugation step, the pellet was washed using 75% ethanol. The air-dried RNA pellet was resuspended in RNAse-free water, and the concentration was determined spectrophotometrically (NanoDrop, Thermo Scientific, Germany).

### 2.5. Microarray Hybridization

Total RNA was incubated with anchored oligo-dT primers at +70°C and subsequently chilled on ice. CDNA synthesis was performed using a mastermix containing first-strand buffer, 20x aminoallyl-dNTP labeling mix, dithiothreitol (DTT), RNAse inhibitor, and reverse transcriptase enzyme (Promega, Germany). Following incubation, residual RNA was degraded by NaOH and HCl incubation. Resulting cDNA was purified using the Qiagen MinElute kit (Qiagen, Germany) and was coupled to Cy3 and Cy5 dyes, respectively, by incubation for 90 min. Cy3-labeled reference and Cy5-labeled sample cDNAs (10 *µ*L each) were combined, denatured by heating for 2 minutes at 98°C, and mixed with 36 *µ*L of hybridization solution at +42°C (Ambion, Austin, TX, USA). Murine microarrays (NimbleGen, Roche, Basel, Switzerland) were overlaid with this solution and hybridized for 18 hours at +42°C using an actively mixing MAUI hybridization system (BioMicro Systems, Salt Lake City, UT, USA). After hybridization, the arrays were washed in 1x SSC/0.05% SDS and 0.1x SSC, centrifuged to remove remaining liquid with unbound cDNA, and air-dried. Intensity values were generated using an array scanner (NimbleGen). Data were uploaded to the mAdb database (Microarray Database, a collaboration of CIT/BIMAS and NCI/CCR at the NIH; URL: http://nciarray.nci.nih.gov/) and formatted via the export function for use with BRB ArrayTools (Biometric Research Branch, NCI, Frederick, MY, USA).

### 2.6. FACS Binding Assay

CAL-1 cells were seeded in 24-well plates one day prior to the experiment and incubated under standard conditions as described above. Fresh cold medium was added together with FITC (5.5 *µ*M), PLarg-complexed 9.2s RNA (2 *µ*g/mL), or FITC-labeled CL264 (5 *µ*g/mL, equaling 5.5 *µ*M) or a combination, respectively (Figure 4(a)). Noncomplexed RNA and PLarg were used as control conditions for 9.2s RNA. Incubation was carried out at +4°C for 10 minutes. Cells suspension was aspired and washed three times with cold PBS, fixed with PFA for 15 minutes at +4°C, and washed another two times with PBS and once with FACS buffer. Cells were then resuspended in 250 *µ*L FACS buffer, followed by readout on a BD FACSCanto II flow cytometer. FACS data was acquired using BD FACSDiva (version 6.1.2) and analyzed using FlowJo (version 10.0.5) software.

### 2.7. Statistics

Data are presented as mean of biological triplicates ± SEM. Each experiment was repeated at least three times. Statistical analysis was performed by two-sided unpaired Student's *t*-test using GraphPad PRISM (La Jolla, CA, USA). The alpha level was set at 5%.

For the analysis of microarray experiments, data from three independent experiments and three untreated controls was used for statistical analyses. Expression analyses were performed using BRB ArrayTools. Data were background corrected, flagged values were removed, spots in which both signals were <100 were filtered out, ratios were log base 2-transformed, and lowest intensity-dependent normalization was used to adjust for differences in labeling intensities of Cy3 and Cy5 dyes [10]. Analysis was restricted to genes present on >50% of the arrays after filtering. The gene expression profile of all treatment groups was compared to that of the control groups. A *p* value cutoff of 0.001 was used to identify genes whose expression was significantly upregulated after TLR7 ligand stimulation when compared to controls. Data were evaluated using Ingenuity Pathway Analysis (IPA, Ingenuity Systems Inc., Redwood City, CA, USA). IPA maps each gene within a global molecular network, pathways, and functional groups developed from information contained in the Ingenuity Pathways Knowledge Base (see URL: https://analysis.ingenuity.com/pa/info/help/Ingenuity_Network_Algorithm_Whitepaper_FINAL(2)).

## 3. Results

### 3.1. Stimulation of CAL-1 Cells with Different TLR7 Ligands: Synergistic Secretion of Proinflammatory Cytokines and Type 1 Interferon

**Figure 1:**
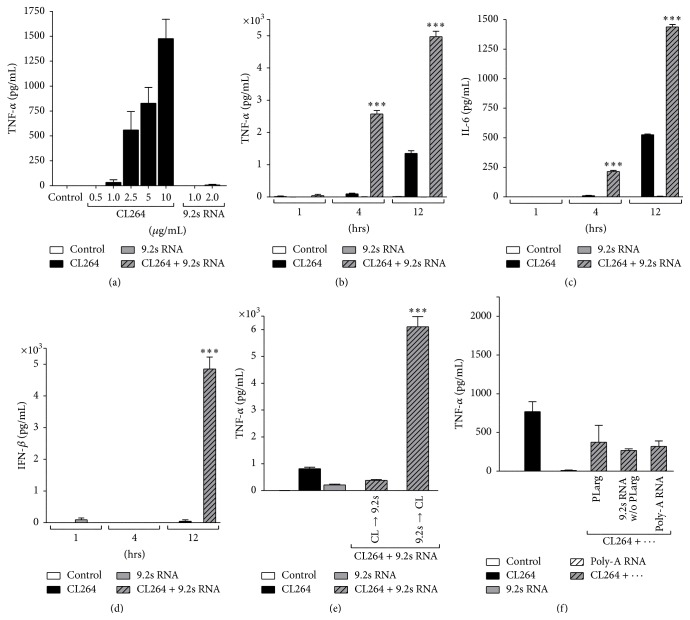
Cytokine and interferon secretion from CAL-1 cells upon stimulation with CL264 and 9.2s RNA. CAL-1 cells were seeded into 96-well plates. After overnight resting, cells were stimulated with CL264 or 9.2s RNA (complexed with PLarg) or the combination of both. After incubation as indicated, cell supernatant was analyzed for TNF-*α*, IL-6, and IFN-*β* by ELISA. (a) Secretion of TNF-*α* upon stimulation with CL264 in concentrations from 0.5 to 10 *µ*g/mL and with 9.2s RNA in concentrations from 1 to 2 *µ*g/mL for 6 hrs. (b) (c) (d) Secretion of TNF-*α* (b), IL-6 (c), and IFN-*β* (d) upon stimulation with CL264 (5 *µ*g/mL), with 9.2s RNA (2 *µ*g/mL) or the combination of both for 1, 4, and 12 hrs. (e) Secretion of TNF-*α* upon sequential stimulation. First, cells were incubated with CL264 (5 *µ*g/mL; left hatched bar) or 9.2s RNA (2 *µ*g/mL; right hatched bar) for 2 hrs, followed by 3 wash steps. After subsequent stimulation with the other ligand for additional 6 hrs, cytokine secretion into the supernatant was measured. (f) Secretion of TNF-*α* upon stimulation with CL264 (5 *µ*g/mL), with 9.2s RNA (2 *µ*g/mL) or with a combination of CL264 and PLarg (2 mg/mL), noncomplexed 9.2s RNA, or nonstimulatory Poly-A RNA (2 *µ*g/mL) for 6 hrs. ^*∗∗∗*^ *p* < 0.005.

CAL-1 cells were stimulated with the specific TLR7 ligand CL264 (adenine analog) and the TLR7/8 ligand 9.2s RNA complexed to PLarg. CAL-1 cells do not express TLR8, so response to 9.2s RNA is limited to TLR7 stimulation [11]. Dose titration experiments determined optimal conditions: using concentrations from 0.5 to 10 *µ*g/mL, stimulation with CL264 resulted in a dose-dependent secretion of TNF-*α* after 6 hours (Figure 1(a)). In contrast, stimulation with 9.2s RNA did not generate any notable response. For further experiments, a submaximal stimulatory concentration of CL264 was used (5 *µ*g/mL). Using this concentration, stimulation with CL264 for up to 12 hours led to a robust, time-dependent release of TNF-*α* reaching absolute levels of 1347 pg/mL TNF-*α* into the supernatant (Figure 1(b)). The kinetics of IL-6 secretion was comparable to that of TNF-*α* with lower absolute cytokine levels (526 pg/mL) after stimulation for 12 hours (Figure 1(c)). In contrast, upon stimulation solely with 9.2s RNA (2 *µ*g/mL), no notable secretion of proinflammatory cytokines was measured. However, the addition of 9.2s RNA to CL264 considerably increased the secretion of TNF-*α* and IL-6 compared to the monostimulation only with CL264 (Figures 1(b) and 1(c);  *p* < 0.001).

We previously demonstrated that activation of CAL-1 cells with TLR9 ligands induces detectable amounts of type 1 IFN [12]. Accordingly, we assessed a possible synergism of TLR7 stimulation on IFN-*β* release under the same experimental conditions using CL264 and 9.2s RNA. Again, costimulation with CL264 and 9.2s RNA for 12 hours resulted in a marked and significant increase of IFN-*β* protein compared to monostimulatory conditions (*p* < 0.001) (Figure 1(d)).

Of note, the synergistic effect of CL264 and 9.2s RNA on CAL-1 cells could be abolished by stimulating the cells sequentially instead of simultaneously. More specifically, no enhanced cytokine secretion could be detected when CL264 was the first stimulus, followed by washing and then ligation with 9.2s RNA. On the other hand, switching the order of stimulation preserved the supra-additive activation even when cells were washed between the ligation steps (Figure 1(e)).

For control experiments, nonstimulatory Poly-A RNA was used instead of 9.2s RNA and had no enhancing effect. Similarly, the use of PLarg alone or 9.2s RNA not complexed with PLarg prior to stimulation had no additive effect (Figure 1(f)).

### 3.2. Changes in Gene Expression Patterns of CAL-1 Cells upon Stimulation with CL264 and/or 9.2s RNA

To gain more insights into the present findings, microarray experiments were performed on CAL-1 cells after stimulation either with CL264, 9.2s RNA, or the combination of both. An early time point for this analysis (4 hrs) was chosen to minimize secondary effects, such as autocrine/paracrine cytokine stimulation. All treatment groups were normalized to untreated controls. Beside the characterization of genes significantly upregulated by either treatment, main goal was the identification of underlying regulatory genes that play a central role for synergistic effects.

**Figure 2:**
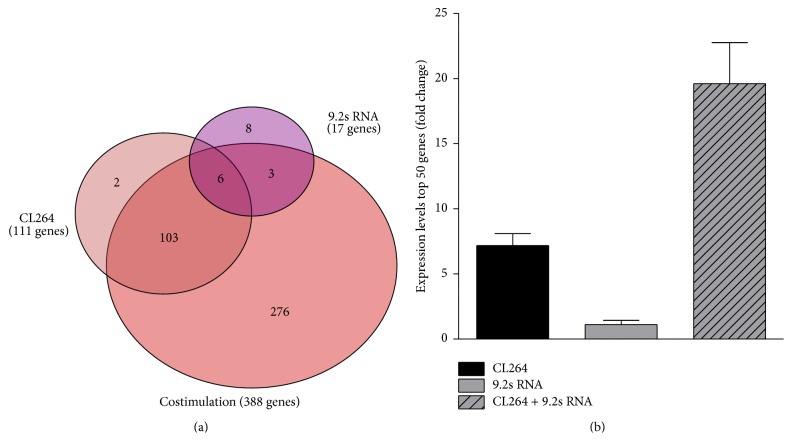
Changes in gene expression patterns of CAL-1 cells upon stimulation with CL264 and/or 9.2s RNA. CAL-1 cells were seeded into 24-well plates. After overnight resting, they were stimulated with CL264 (5 *µ*g/mL) or 9.2s RNA (2 *µ*g/mL) or the combination of both. After incubation for 4 hrs, cells were lysed and total RNA was extracted. Following cDNA synthesis, labeled cDNA was hybridized to microarrays. Treatment groups were normalized to untreated controls. (a) Venn diagram showing changes in gene expression patterns using a statistical cutoff of *p* < 0.001. (b) Expression levels of the 50 most significantly upregulated genes in each treatment group, compared to untreated cells.

Using a statistical cutoff of *p* < 0.001, treatment with 9.2s RNA significantly increased expression of 17 genes in CAL-1 cells, while treatment with CL264 resulted in an upregulation of 111 genes (Figure 2(a)). However, costimulation with both TLR7 ligands resulted in a synergistic upregulation of 388 genes, thereby upregulating significantly more genes than the sum of genes upregulated by either ligand alone. Interestingly, the majority of the upregulated genes in the monostimulatory groups (*n* = 112, 92%) were also present in the costimulatory group. Furthermore, we addressed the question whether the unique genes significantly upregulated solely by costimulation (*n* = 276, 71%) were also increased in the monostimulatory groups when the statistical cutoff was lowered by one magnitude (*p* < 0.01). In this case, 96 additional genes could be ascribed to either of the monostimulatory groups. Altogether, 208 genes (54% out of 388 genes; *p* < 0.01) in the costimulatory group were significantly upregulated in either of the monostimulatory groups. Overall, these results indicate that the synergistic effect found in the costimulatory group is partially due to a stronger stimulation of already regulated genes but furthermore an effect of the upregulation of so far unregulated genes.

We next addressed the question whether costimulation with CL264 and 9.2s RNA also resulted in a significant increase of gene activation in terms of magnitude. Therefore, we analyzed the expression levels of the 50 most significantly upregulated genes in each treatment group. Results show that the average fold change increased from 1.11 (9.2s RNA) and 7.17 (CL264) to 19.6 (9.2s RNA/CL264) (Figure 2(b)) compared to untreated cells. Together, these results indicate that costimulation with 9.2s RNA and CL264 resulted in a synergistic effect on gene expression in terms of both number and magnitude of upregulated genes compared to monostimulation.

### 3.3. Analysis of Pathways, Functional Groups, and Regulatory Networks Triggered by 9.2s RNA Plus CL264

IPA was used to identify significantly regulated canonical pathways and functional groups. IPA characterizes gene products based on their function and role in regulatory pathways. Top scoring canonical pathways were “*activation of IRF by cytosolic pattern recognition receptors*” (*p* < 4.31*E* − 13), “*dendritic cell maturation”* (*p* < 1.08*E* − 10),* and “interferon signaling*” (*p* < 5.94*E* − 10). Top scoring functional groups were, for example, “*cellular development, growth, and proliferation*” (*p* < 7.26*E* − 34), “*cell death and survival*” (*p* < 5.72*E* − 29), “*infectious disease*” (*p* < 1.6*E* − 24), and “*inflammatory response*” (*p* < 1.2*E* − 20) (for more see Tables 1 and 2). These results indicate that the synergistic effect especially refers to immune-related processes.

**Figure 3:**
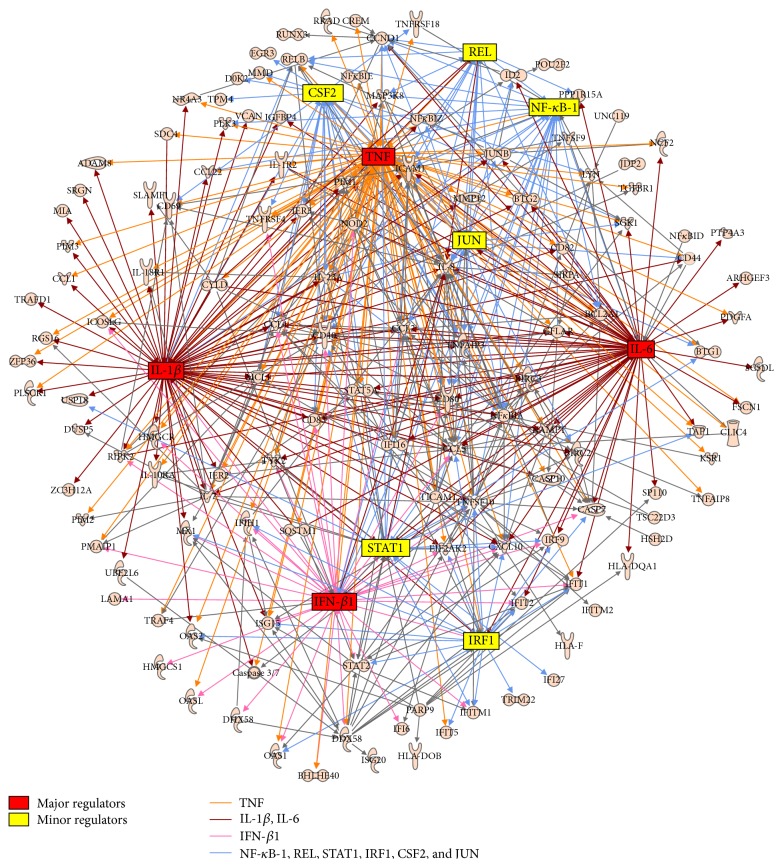
Analysis of regulatory networks triggered by 9.2s RNA plus CL264. CAL-1 cells were seeded into 24-well plates. After overnight resting, they were stimulated with CL264 (5 *µ*g/mL) and 9.2s RNA (2 *µ*g/mL). After incubation for 4 hrs, cells were lysed and total RNA was extracted. Following cDNA synthesis, labeled cDNA was hybridized to microarrays. The network analysis identifies major (red) and minor regulatory nodes (yellow) of the synergistic regulation.

Furthermore, IPA was used to identify the pattern of regulatory interactions underlying synergistic gene activation after costimulation with 9.2s RNA and CL264. The network analysis enabled us to define the regulatory nodes of this synergistic regulation. Major regulators were previously defined as regulatory nodes that trigger more than 30% of the costimulatory network, while minor regulators were defined as regulating 10% to <30% of the network [13, 14]. Applying this analysis revealed IL-1*β*, IL-6, and IFN-*β* as major regulatory nodes, while several minor regulatory nodes were also identified (NF-*κ*B-1, REL, STAT1, CSF2, IRF1, and JUN) (Figure 3). Together, the major regulators (IL-1*β*, IL-6, TNF, and IFN-*β*) impacted the expression of around 95% of all genes within the network of the costimulatory group, while the minor regulators only had modulatory properties on the induced gene expression network.

### 3.4. Identification of Genes Synergistically Upregulated by 9.2s RNA Plus CL264

Evidence that the combination of CL264 and 9.2s RNA synergistically enhanced the secretion of certain cytokines and induced broader networks of gene regulation led us to examine which specific genes were synergistically upregulated by the combination of 9.2s RNA and CL264. Therefore, genes in the costimulated group with expressions being upregulated 1.5-fold of the sum induced by the ligands 9.2s RNA and CL264 alone were defined as being synergistically regulated. 62 genes were identified to be synergistically regulated by this criterion (Table 3). Out of these genes, 65% were functionally related to “*immune response*” regulation and 60% were related to “*cellular growth and proliferation.*” Furthermore, three out of four major regulators (see above, Figure 3) belonged to the synergistically regulated genes. Other synergistically regulated genes included the cytokines IL-1b, IL-6, IL-18R, and IL-23A, the chemokines CCL1, CCL2, CXCL8, and CXCL10, and the CD genes CD40, CD44, CD69, CD70, and CD83 (Table 3), again confirming that the synergistic effect especially refers to immune-related processes.

### 3.5. Coincubation with 9.2s RNA Enhances Binding of CL264 to CAL-1 Cells

**Figure 4:**
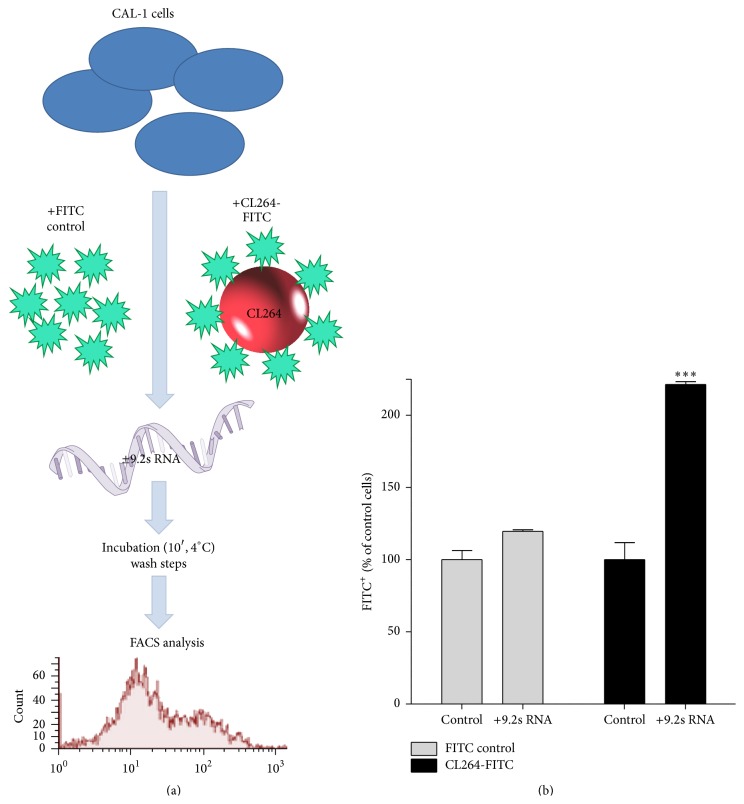
Enhanced binding of CL264 to CAL-1 cells upon costimulation with 9.2s RNA. (a) CAL-1 cells were seeded into 24-well plates. After overnight resting, they were incubated with FITC control (5.5 *µ*M), FITC-labeled CL264 (5 *µ*g/mL, equaling 5.5 *µ*M), or PLarg-complexed 9.2s RNA (2 *µ*g/mL) or a combination for 10 minutes at +4°C. After extensive washing and fixing with PFA, cells were resuspended in FACS buffer, followed by readout on a flow cytometer. (b) Proportion of FITC-positive cells (expressed as percentage of cells incubated solely with FITC control). Combination with 9.2s RNA significantly enhances binding of FITC-CL264 to the cell surface. ^*∗∗∗*^ *p* < 0.005.

To gain a more functional insight into the synergistic effect of CL294 and 9.2s RNA, we finally performed surface binding studies, since microarray analysis suggested that CL264 had a stronger impact on CAL-1 cells when being coadministered with 9.2s RNA. Therefore, CAL-1 cells were incubated with FITC-labeled CL264 alone or in combination with 9.2s RNA. The binding to the cell surface was determined by FACS analysis (Figure 4(a)). Cells being incubated with 9.2s RNA and FITC as control showed no increase in fluorescence signaling compared to the incubation with FITC alone. However, the combination of 9.2s RNA significantly enhanced the binding of FITC-labeled CL264 to the surface of the cells after a 10-minute stimulation period compared to the FITC control (221%, *p* < 0.001, Figure 4(b)). RNA not being complexed to PLarg or PLarg alone used as controls had no enhancing effect on the binding of labeled CL264.

## 4. Conclusions

TLRs are essential for host immunity by recognizing conserved structures in pathogens which trigger innate immune responses and prime antigen-specific adaptive immunity [1]. Among the nucleic acid-sensing TLRs 3, 7, and 9, all being located intracellularly, TLR7 and TLR9 share many common characteristics [15–17]. TLR7 and TLR9 ligands have shown promise in clinical trials as immunotherapeutics for the treatment of cancer, allergy, and infectious diseases [6, 18, 19]. By utilizing the human pDC line CAL-1, we were able to demonstrate a so far undescribed synergistic immune activation by the simultaneous use of CL264 and 9.2s RNA. In particular, costimulation with both ligands resulted in a supra-additive induction of proinflammatory cytokines (IL-6 and TNF-*α*) as well as type 1 IFN (Figure 1). Microarray analysis showed that simultaneous use of both ligands synergistically activated genes primarily associated with immune processes (Figure 3). Mechanistically, surface binding studies revealed the enhanced uptake of CL264 in the presence of 9.2s RNA (Figure 4), which could account for the observed synergism.

PDCs were chosen for this study because they respond to TLR7 and are key players linking the innate and adaptive immune response. Of note, the use of human primary pDCs is inherently difficult for two reasons; first, pDCs are present in very low frequency in human peripheral blood and lymphoid tissue, accounting for approximately 0.2–0.5% of PBMC [20]; second, any efforts in purifying pDCs are likely to activate (prime) the cells, which contorts pattern of subsequent stimulation. As an alternative, the human pDC-derived CAL-1 line was studied. This cell line shares many of the phenotypic and functional properties of freshly isolated human pDCs and mirrors their response to TLR stimulation [9, 11, 21].

We stimulated our cells simultaneously with two different ligands for TLR7. Similarly to the substituted 8-hydroxyadenine derivative SM360320, CL264 induces the activation of NF-*κ*B and the secretion of type 1 IFN in TLR7-expressing cells [22]. CL264 ligand activity is TLR7-specific, it does not stimulate TLR8 even at high concentrations (>10 *µ*g/mL). The immunostimulatory properties of the single-stranded RNA 9.2s were first described in 2005, when Hornung et al. originally intended to design different siRNAs (named 9.1 to 9.4) downregulating the human TLR9 to avoid effects of non-target-related IFN induction in human PDCs [8]. In fact, transfection of HEK293 cells with these RNAs reduced TLR9 expression. However, when the authors of that study transfected human pDCs using these siRNAs, they surprisingly noted a consistent pattern of type 1 IFN production, with siRNA 9.2 being the strongest inducer of IFN production. Additional analyses revealed that a specific sequence of nine bases at the 3′ end (*5*′*-GUCCUUCAA-3*′) of the sense strand of siRNA9.2 (termed RNA 9.2s, in contrast to 9.2a, which stands for the antisense strand) was responsible for the immunostimulatory activity. Injection of 9.2 RNA into TLR7-deficient mice elicited, in contrast to the injection into wild-type mice, no detectable IFN serum levels.

Microarrays facilitate the evaluation of global changes in gene expression induced by immune stimuli [23–25]. This has led others to use microarray approaches to monitor global changes of gene expression induced by the combination of several inflammatory stimuli [26–29]. In general, almost all studies observed synergistic upregulation of immune-related genes when combining ligands for different TLRs [27, 29]. Rationale is always the simultaneous activation of different pathways inducing a supra-additive immune activation. However, we opted for a slightly different approach by creating synergistic effects using structurally different ligands targeting the same receptor. We observed a more than 3-fold increase in the number of genes upregulated by the combination of CL264 and 9.2s RNA compared to CL264 alone (Figure 2(a)). Additionally, the overall magnitude of gene upregulation was increased by more than 2.5-fold when comparing the top 50 genes induced upon combination of both ligands versus CL264 alone (Figure 2(b)). Studies of protein production by ELISA confirmed the synergistic activation of regulatory members such as IL-6, TNF, and IFN-*β* (Figure 1). These findings suggest that simultaneous exposure to both ligands magnified and accelerated the response elicited by each individual ligand. Importantly, the vast majority of genes upregulated by stimulating with either ligand alone were also induced in the costimulatory setting (92%). Investigating the genes upregulated in the combined CL264/9.2s RNA group, nearly 54% of them were also upregulated in the monostimulatory settings (although at a lower *p* value). Taken together, this indicates a stronger stimulation of genes yet upregulated upon stimulation by either ligand. On the other hand, this finding also delineates the upregulation of new or rather so far unregulated genes by the combination of CL264/9.2s RNA (46% of the genes). These synergistic effects primarily broaden the existing immune response, rather than creating a new one. This observation is supported by the fact that the functional groups (analyzed by IPA) induced by the combinatory versus single stimulation did not alter qualitatively (*innate immune function* such as IRF signaling or DC maturation (Table 2)).

Finally, exclusive IPA analysis of the 62 synergistically upregulated genes (defined as upregulated 1.5-fold of the sum induced by the ligands 9.2s RNA and CL264 alone; see Table 3) revealed that 65% were immune-related. Indeed, more than 35% of all synergistically upregulated genes, including the most highly upregulated, coded for cytokines, chemokines, or transcription regulators (such as IL-1b, IL-6, IL-18R, IL-23A, CCL1, CCL2, REL, and NFKBIZ). These findings support the hypothesis that stimulation via different TLR7 ligands synergistically broadens and increases the magnitude of the host's protective immune response. A similar observation has recently been made by Du et al., who showed that the combination of small phosphothioate ODNs with the TLR7/8 agonist CL075 (a chemical base analog comparable to CL264) induced a synergistic response in murine glial cells with substantially higher levels of proinflammatory cytokines and chemokines compared to CL075 [30]. Mechanistically, that work suggested intracellular processes underlying these synergistic effects. However, we identified another possible cause. Using a FACS-based binding assay, we showed increased cell surface binding (>2-fold) of CL264 upon combination with 9.2s RNA compared to CL264 alone (Figure 4). By stimulating the cells sequentially, 9.2s RNA could be identified as being accountable for this phenomenon (Figure 1(f)). Although the mechanistic background remains to be elucidated, this finding suggests an optimized surface binding and subsequent cellular uptake into the endolysosomal compartment as one explanation for the observed synergistic effects of CL264 and 9.2s RNA. Of note, this effect is not due to the use of the cationic polypeptide Plarg together with 9.2s RNA, since synergy was not observed when CL264 was administered with Plarg alone. Further investigations are necessary to clarify whether binding is limited at the cell surface or leads to enhanced localization of CL264 within the endosomes in the presence of 9.2s RNA, maybe using confocal microscopy.

We cannot rule out further additive effects on the receptor level using both types of ligands. With regard to the abovementioned properties of 9.2s RNA, our microarray data do not show any evidence for a specific influence of 9.2s RNA on the closely related TLR9 pathway (data not shown). Since CAL-1 cells do not express the TLR8, costimulatory effects of TLR7 and TLR8 by the use of ssRNA and CL264 are highly improbable [11]. However, Tanji et al. very recently showed two distinct binding sites of ssRNA fragments and chemical ligands at TLR8 leading to synergistic effects when being simultaneously activated [31]. Since TLR7 and TLR8 are closely related and recognize the same ligands, it is conceivable that the same phenomenon also accounts for TLR7. Thus, optimal stimulation of TLR7 at different binding sites by ssRNA and base analogs could also contribute to the observed synergistic effects of this study. Upcoming experiments are designed to gain more mechanistic insights into the intracellular level underlying these effects by combining different ssRNA motifs and CL264.

Although various synthetic TLR7/8 agonists have been used as adjuvants during preclinical trials, not many of them are approved for use in humans. The TLR7/8 agonist R837 is used for the topical treatment of genital warts, basal cell carcinoma, and bladder cancer [32]. However, systemic application of imidazoquinolines causes adverse side effects, and the development of other TLR7 agonists suitable for nontopical use as adjuvants is desirable [6, 33].

Our study has some limitations. Despite the fact that CAL-1 cells share many of the phenotypic and functional properties of human pDCs, Maeda et al. have described some significant differences between this cell line and freshly isolated primary human pDCs including little IFN-*α* secretion in response to TLR stimulation [11]. It is well accepted that working with purified human pDCs is challenging, since they are extremely sensible to any kind of physical manipulation (such as purifying them using different sorting techniques), resulting in significant cytokine release acting in a paracrine and autocrine fashion [20, 34]. Kim et al. recently demonstrated that low levels of background IFN-*β* (so-called primed pDCs) resulted in a strong feedforward regulation of their TLR-triggered type 1 IFN production* in vitro* and concluded that the* in vitro* activity of a TLR ligand on primary pDCs does not necessarily correspond to its* in vivo* activity [35]. However, we and many other groups have meanwhile shown that CAL-1 cells are suitable surrogates to study specific aspects of human pDC physiology and TLR7 and TLR9 [9, 12, 36–38]. For the herein presented study, we chose to focus on IFN-*β* (instead of IFN-*α*) since we studied early time point effects up to 12 hours to minimize the mentioned auto- and paracrine cytokine effects, which is essential when studying synergism of different TLR ligands. We previously demonstrated that, in contrast to IFN-*β*, IFN-*α* expression peaks rather late (>24 hours) in response to TLR stimulation, which is likewise influenced by a type 1 IFN feedback loop [12].

A number of studies analyzed synergistic effects activating different TLRs [26–29]. However, to our knowledge, the present study is the first to provide fundamental new insights into the simultaneous activation of a single toll-like receptor. Using the human pDC-like cell line CAL-1, we demonstrated that structurally different TLR7 ligands act synergistically on gene expression patterns of the inflammatory response. This phenomenon may be explained in part by an enhanced binding of CL264 in the presence of 9.2s RNA. However, future studies will need to further elucidate intracellular mechanisms, as suggested by recent studies [31]. Taken together, our data could impact future strategies optimizing TLR7-targeted drug design and provide new insights into the synergistic interactions of structurally different TLR7 ligands.

## Figures and Tables

**Table 1:** Top 5 canonical pathways (signaling): costimulation for 4 hrs (CL264 and RNA).

Pathway	*p* value
Activation of IRF by cytosolic pattern recognition receptors	<4.31*E* − 13
Dendritic cell maturation	<1.08*E* − 10
Interferon signaling	<5.94*E* − 10
Communication between innate and adaptive immune cells	<1.05*E* − 08
Death receptor signaling	<1.2*E* − 08

IRF: interferon regulatory factor.

CAL-1 cells were seeded into 24-well plates. After overnight resting, they were stimulated with CL264 (5 *µ*g/mL) or 9.2s RNA (2 *µ*g/mL) or the combination of both. After incubation for 4 hrs, cells were lysed and total RNA was extracted. Following cDNA synthesis, labeled cDNA was hybridized to microarrays. Treatment groups were normalized to untreated controls. Table lists the 5 most upregulated canonical pathways as identified by Ingenuity Pathway Analysis (IPA).

**Table 2:** Top 10 functional groups.

Functional group	*p* value
Cellular development, growth, and proliferation	<7.26*E* − 34–6.8*E* − 07
Hematological system development and function	<7.26*E* − 34–6.8*E* − 07
Cellular function and maintenance	<5.44*E* − 31–1.93*E* − 07
Cell death and survival	<5.72*E* − 29–6.36*E* − 07
Tissue morphology	<2.58*E* − 26–4.79*E* − 07
Infectious disease	<1.60*E* − 24–4.55*E* − 07
Immunological disease	<8.8*E* − 22–4.05*E* − 07
Cell-to-cell signaling and interaction	<1.20*E* − 20–5,89*E* − 7
Immune cell trafficking	<1.20*E* − 20–5,89*E* − 07
Inflammatory response	<1.20*E* − 20–6,74*E* − 07

CAL-1 cells were treated as described above (see Table 1). The table lists the 10 most upregulated functional groups as identified by IPA.

**Table 3:** Genes synergistically upregulated by the combination of 9.2s RNA and CL264.

Gene name	Unigene ID	Gene symbol	Transcript level (fold over nonstimulated control)			
			9.2s RNA	CL264	9.2s RNA + CL264	Synergistic increase
CD69 molecule	Hs.208854	CD69	2.4	6.1	64.2	7.6
BCL2-related protein A1	Hs.227817	BCL2A1	2.7	22.2	96.6	3.9
baculoviral IAP repeat containing 3	Hs.127799	BIRC3	1.2	6.4	25.6	3.4
RALBP1 associated Eps domain containing 2	Hs.186810	REPS2	1.0	2.4	10.3	3.1
Interferon, beta 1, fibroblast	Hs.93177	IFNB1	1.6	1.9	10.8	3.1
Suppressor of tumorigenicity 20	Hs.729127	ST20	1.3	4.8	18.5	3.0
Chemokine (C-C motif) ligand 2	Hs.303649	CCL2	0.6	41.4	126.0	3.0
CD44 molecule	Hs.502328	CD44	1.5	4.3	17.5	3.0
Early growth response 3	Hs.534313	EGR3	2.1	3.3	15.9	2.9
Tubulin tyrosine ligase-like family	Hs.520554	TTLL2	1.1	3.1	11.9	2.8
Oligonucleotide/oligosaccharide-binding fold containing 2A	Hs.591610	OBFC2A	1.7	12.4	38.6	2.7
Interleukin 6 (interferon, beta 2)	Hs.654458	IL-6	0.9	4.9	14.7	2.6
Nuclear factor of kappa light polypeptide gene enhancer in B cells inhibitor, alpha	Hs.81328	NFKBIA	1.6	6.2	19.6	2.5
MIR155 host gene (non-protein-coding) (MIR155HG), noncoding RNA.	N/A	MIR155HG	1.5	1.9	8.1	2.4
Chemokine (C-C motif) ligand 1	Hs.72918	CCL1	2.4	15.6	41.8	2.3
RasGEF domain family, member 1B	Hs.591696	RASGEF1B	1.1	3.3	10.2	2.3
CD70 molecule	Hs.501497	CD70	1.4	6.1	16.5	2.2
G protein-coupled receptor 183	Hs.784	GPR183	1.0	2.6	7.8	2.2
ADAM metallopeptidase with thrombospondin type 1 motif, 10	Hs.657508	ADAMTS10	1.0	1.9	6.1	2.1
ADAM metallopeptidase domain 19	Hs.483944	ADAM19	2.4	3.6	12.6	2.1
FSHD region gene 2 family, member C	Hs.274541	FRG2C	0.8	1.1	4.1	2.1
Interleukin 23, alpha subunit p19	Hs.98309	IL-23A	1.6	7.8	19.4	2.1
EF-hand calcium binding domain 3	Hs.152670	EFCAB3	1.0	1.3	4.8	2.1
Neutrophil cytosolic factor 2	Hs.587558	NCF2	1.8	3.8	11.5	2.1
Interleukin 1, beta	Hs.126256	IL-1B	1.0	7.3	17.0	2.1
Nuclear receptor subfamily 4, group A, member 3	Hs.279522	NR4A3	1.4	3.1	9.4	2.1
Tumor necrosis factor, alpha-induced protein 3	Hs.211600	TNFAIP3	1.5	3.5	10.2	2.1
Regulator of G-protein signaling 1	Hs.75256	RGS1	1.7	3.5	10.7	2.0
Serum/glucocorticoid regulated kinase 1	Hs.510078	SGK1	0.5	1.8	4.8	2.0
v-rel reticuloendotheliosis viral oncogene homolog	Hs.631886	REL	1.1	2.4	6.7	2.0
Immediate early response 3	Hs.76095	IER3	0.8	4.3	9.9	1.9
Nuclear factor of kappa light polypeptide gene enhancer in B cells inhibitor, zeta	Hs.319171	NFKBIZ	1.3	2.7	7.8	1.9
Chemokine (C-C motif) ligand 4-like 2	Hs.661942	CCL4L2	2.0	21.0	43.9	1.9
Interleukin 18 receptor 1	Hs.469521	IL-18R1	2.5	6.9	17.9	1.9
Interleukin 8 (IL8)	Hs.624	IL-8	1.8	2.4	7.9	1.9
CASP8 and FADD-like apoptosis regulator	Hs.390736	CFLAR	1.5	2.2	6.8	1.9
pim-2 oncogene	Hs.496096	PIM2	0.8	3.0	7.1	1.8
Spermidine/spermine N1-acetyltransferase 1	Hs.28491	SAT1	1.6	3.6	9.5	1.8
Interleukin 4 induced 1	Hs.574492	IL-4I1	1.2	4.0	9.4	1.8
Chemokine (C-X-C motif) ligand 10	Hs.632586	CXCL10	1.6	5.8	13.1	1.8
Solute carrier family 29 (nucleoside transporters), member 2	Hs.569017	SLC29A2	1.4	3.4	8.6	1.8
Long intergenic non-protein-coding RNA 173, noncoding RNA.	N/A	LINC00173	1.8	3.2	8.8	1.8
Mitogen-activated protein kinase kinase kinase 8	Hs.432453	MAP3K8	1.1	4.1	9.1	1.8
Signal transducer and activator of transcription 5A	Hs.437058	STAT5A	0.8	1.9	4.6	1.7
ADP-ribosylation factor-like 5B	Hs.25362	ARL5B	1.5	3.1	7.9	1.7
Ninjurin 1	Hs.494457	NINJ1	0.9	3.4	7.3	1.7
Wingless-type MMTV integration site family, member 10A	Hs.121540	WNT10A	1.2	5.8	11.6	1.6
B cell translocation gene 1, antiproliferative	Hs.255935	BTG1	1.5	2.6	6.7	1.6
Fascin homolog 1, actin-bundling protein (*Strongylocentrotus purpuratus*)	Hs.118400	FSCN1	0.5	1.6	3.5	1.6
CD83 molecule	Hs.595133	CD83	1.7	5.2	11.4	1.6
Platelet-derived growth factor alpha polypeptide	Hs.535898	PDGFA	1.4	2.3	6.1	1.6
Transcription factor EC	Hs.125962	TFEC	0.6	3.1	6.1	1.6
CD40 molecule, TNF receptor superfamily member 5	Hs.472860	CD40	1.0	4.1	8.4	1.6
POU class 2 homeobox 2	Hs.654420	POU2F2	0.6	3.2	6.2	1.6
Nuclear receptor coactivator 7	Hs.171426	NCOA7	1.6	2.7	6.8	1.6
Zinc finger protein 36, C3H type-like 1	Hs.85155	ZFP36L1	1.3	2.0	5.1	1.6
Solute carrier family 7, member 11	Hs.390594	SLC7A11	1.4	2.2	5.7	1.6
FSHD region gene 2	Hs.626907	FRG2	1.1	1.3	3.7	1.5
Homo sapiens microRNA miR-146 stem-loop	N/A	hsa-mir-146a	1.1	3.7	7.3	1.5
Homo sapiens microRNA miR-21 stem-loop	N/A	hsa-mir-21	1.6	1.7	5.1	1.5
BTB (POZ) domain containing 19	Hs.632400	BTBD19	0.8	1.7	3.9	1.5
ELOVL fatty acid elongase 6	Hs.412939	ELOVL6	1.1	1.7	4.2	1.5

CAL-1 cells were treated as described above (see Table 1). The table lists specific genes synergistically upregulated by the combination of 9.2s RNA and CL264. Genes in the costimulated group with expressions being upregulated 1.5-fold of the sum induced by the ligands 9.2s RNA and CL264 alone were defined as being synergistically regulated.
